# The expression and significance of P-glycoprotein, lung resistance protein and multidrug resistance-associated protein in gastric cancer

**DOI:** 10.1186/1756-9966-28-144

**Published:** 2009-11-24

**Authors:** Wen-Qing Hu, Chun-Wei Peng, Yan Li

**Affiliations:** 1 Department of Surgery, Heji Hospital Affiliated to Changzhi Medical College, Changzhi 046011, PR China; 2 Department of Oncology, Zhongnan Hospital of Wuhan University, Hubei Key Laboratory of Tumor Biological Behaviors & Hubei Cancer Clinical Study Center, Wuhan 430071, PR China

## Abstract

**Background:**

To detect the expression of multidrug resistance molecules P-glycoprotein (P-gp), Lung resistnce protein (LRP) and Multidrug resistance-associated protein (MRP) and analyze the relationship between them and the clinico-pathological features.

**Methods:**

The expressions of P-gp, LRP and MRP in formalin-fixed paraffin-embedded tissue sections from 59 gastric cancer patients were determined by a labbelled Streptavidin-Peroxidase (SP) immunohistochemical technique, and the results were analyzed in correlation with clinicopathological data. None of these patients received chemotherapy prior to surgery.

**Results:**

The positive rates of P-gp, LRP, MRP were 86.4%, 84.7% and 27.1%, respectively. The difference between the positive rate of P-gp and MRP was significant statistically, as well as the difference between the expression of MRP and LRP. No significant difference was observed between P-gp and LRP, but the positively correlation between the expression of P-gp and LRP had been found. No significant correlation between the expression of P-gp, LRP, MRP and the grade of differentiation were observed. The expression of P-gp was correlated with clinical stages positively (r = 0.742), but the difference with the expression of P-gp in different stages was not significant.

**Conclusion:**

The expressions of P-gp, LRP and MRP in patients with gastric cancer without prior chemotherapy are high, indicating that innate drug resistance may exist in gastric cancer.

## Background

Gastric cancer (GC) is one of the most common malignancies worldwide. Despite noticeable advancements in the prevention, diagnosis and treatment, GC still accounts for over 10% of global cancer mortality, and remains the second most frequent cause of cancer death after lung cancer [1,2], while in Asia, it is the top killing cancer [3]. Among the estimated 934,000 GC new cases and 700,000 GC deaths in 2002, China alone accounts for almost 42% of the global total, with age-standardized incidence rates of 41.4/100,000 for males and 19.2/100,000 for females [2]. A recent national survey in China shows that GC is the No 3 cancer killer after lung cancer and liver cancer, with 24.71/100,000 death rate [4].

Current major treatment modalities for GC include surgery and chemotherapy/radiotherapy. Curative gastrectomy with proper loco-regional lymph node dissection is the treatment of choice for resectable GC [5]. The effects of chemotherapy for GC are limited because multidrug resistance (MDR) problem in the primary tumor usually leads to treatment failure. There are quite a number of different mechanisms accounting for drug resistance, and MDR protein family plays an essential role. MDR refers to subsequent and cross-over resistance to drug of different categories, after exposure of tumor to a chemotherapeutic agent [6]. Currently, the over expressions of P-glycoprotein (P-gp), Multidrug resistance-associated protein (MRP) and Lung resistnce protein (LRP) are most extensively studied in MDR. Using immunohistochemical technique, this study was to determine the protein expressions of P-gp, LRP and MRP in GC tissues from patients without chemotherapy, and explored their expressions with clinico-pathological factors.

## Materials and methods

### Patients and tissue samples

GC specimens from 59 patients without prior chemotherapy were collected from HeJi Hospital affiliated to Changzhi Medical College from January 2001 to December 2003. All tumors were fixed with formalin and embedded with paraffin. There were 46 (78.0%) males and 13 (22.0%) females with the median age of 55 years (range: 32~75 years). Pathological diagnoses were poorly differentiated adenocarcinoma in 18 cases (30.5%), moderately differentiated adenocarcinoma in 23 cases (39.0%), well differentiated adenocarcinoma in 8 cases (13.6%), mucous adenocarcinoma in 6 cases (10.2%) and unknown pathological type in 4 cases (6.8%).

### Regents

The reagents used in this study were rabbit anti-MRP1 (bs-0657R, 1:300 dilution), rabbit anti-pGP/MDR1/gp170 (bs-0563R, 1:300 dilution), rabbit anti-LRP (bs-0661R, 1:300 dilution) and Biotin conguated Goat Anti-rabbit IgG, all obtained from Beijing Biosynthesis Biotechnology Corporation (Beijing, China). Bovine serum albumin (BSA, 2%), IHC Biotin Block Kit, Streptavidin-Peroxidase and diaminobenzidine (DAB) were from Fujian Maixin Biotechnology Corporation (Fuzhou, China).

### Immunohistochemistry

Immunolocalization of MDR markers were performed according to the streptavidin-biotin peroxidase complex method by Truong [7]. Tissue slides were first deparaffinized in xylol, ethanol, and water, and then endogenous peroxidase activity was blocked by immersion in 3% H_2_O_2 _in methanol for 10 min to prevent any nonspecific binding. For staining, the slides were pretreated in 0.01 M citrate buffer (pH 6.0) and heated in a microwave oven (98°C) for 10 min. After blocking with BSA, the slides were incubated with the primary antibodies for P-gp, LRP and MRP for 90 min at 37°C, then incubated with the secondary antibody (biotin-labeled anti-rabbit IgG goat antibody) for 15 min at 37°C, and finally incubated with peroxidase-labeled streptavidin for 15 min. The reaction products were visualized with diaminobenzidine.

Positive cells were stained brownish granules. Ten high power fields in each slide were selected randomly and observed double blind by two investigators. The staining score of each section were calculated by staining intensity and positive rate of cancer cells. For the quantification of staining intensity, the score of no staining, weak staining, moderate staining and strong staining was 0, 1, 2 and 3 respectively. Positive rate score of cancer cells was: 0-10% was recorded as 0; 10-30% was recorded as 1; 30-50% was recorded as 2; 50-75% were recorded as 3; >75% were recorded as 4. The sum of scores was computed as the score of staining intensity added the score of the positive rate of cancer cells. Then it was graded according the sum of scores: 0-1 (-); 2-3 (+); 4-5 (++); 6-7 (+++).

### Statistical Analysis

All the experiment data is integrated into a comprehensive data set. Numerical data were recorded directly and measurement data were described as median and range. We analyzed categorical variables using the Pearson Chis-square test and Gamma test. Statistical analysis was performed on SPSS software version 13.0 (SPSS Inc. Chicago, IL), and P < 0.05 was considered as statistically significant.

## Results

### Location and distribution of P-gp, LRP and MRP

**Figure 1:**
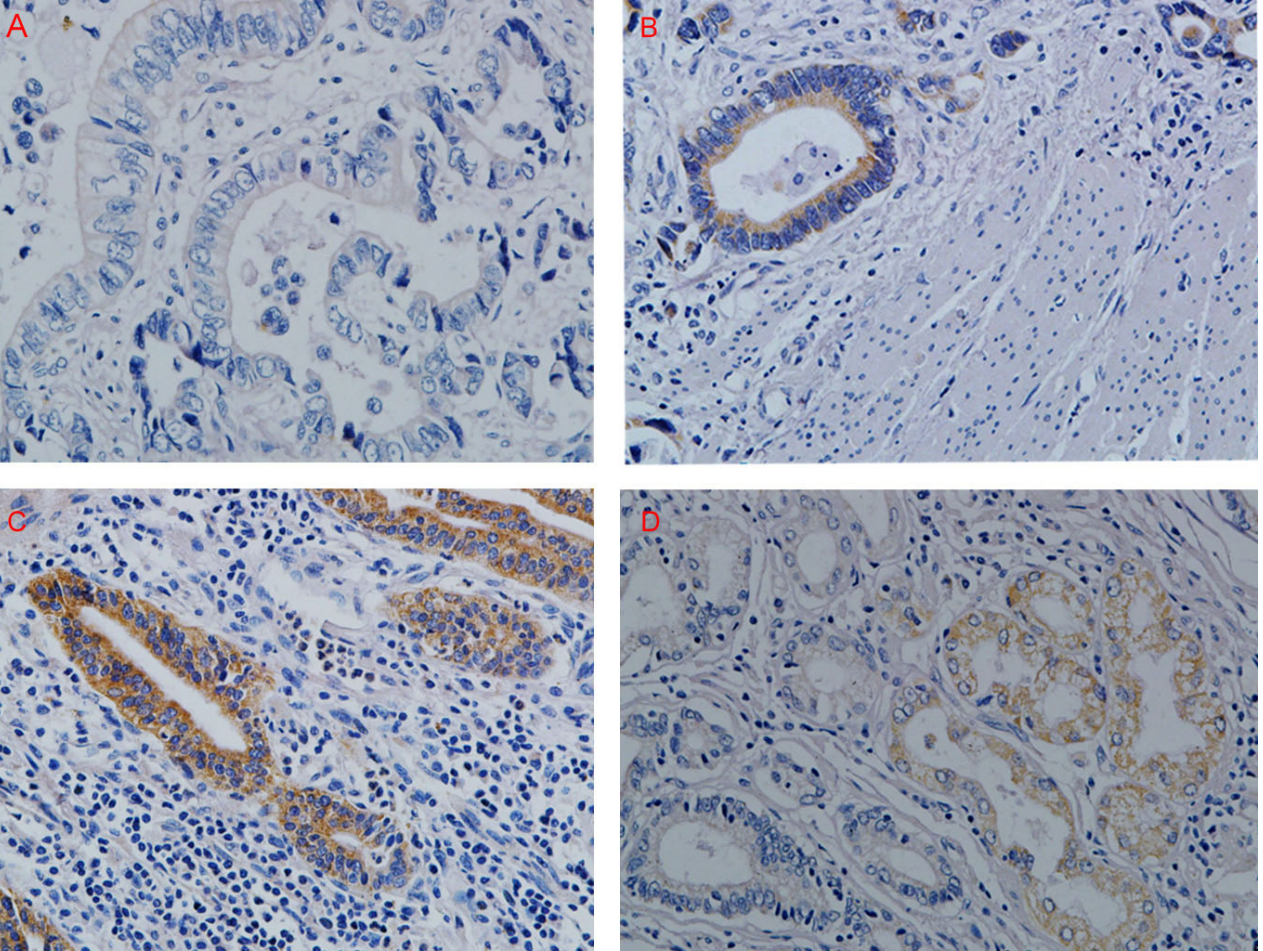
**The expression of P-gp (B), LRP (C) and MRP (D) in gastric cancer tissues**. A. Negative control; B. IHC detection of P-gp; C. IHC detection of LRP; D. MRP detection of MRP. All with hematoxylin background staining (× 400).

There was a clear background without nonspecific staining in negative control slides (Fig 1A). The three proteins were stained brownish granules, with P-gp mainly located on the membrane and cytoplasm (Fig 1B), LRP on peri-nuclear cytoplasm (Fig 1C), and MRP on the membrane and cytoplasm (Fig 1D). The characteristic distribution pattern of three proteins was scattered expression in tumor tissue, although small areas of diffused expression were also observed.

### The expression of P-gp, LRP and MRP

In the 59 cases, the positive rate of P-gp (86.4%) was significantly higher than MRP (27.1%) (*P *= 0.000). No significant difference between the expression of P-gp (86.4%) and LRP (84.7%) were observed (*P *= 1.000), but we found the positive correlation between them (r = 0.803). The positive rate of LRP (84.7%) was significantly higher than MRP (27.1%) (*P *= 0.000) (Table 1).

**Table 1:** The Expression of P-gp, MRP and LRP in 59 cases with gastric cancer

	expression**				
		
MDR proteins*	--- n (%)	+ n (%)	++ n (%)	+++ n (%)	Positive numbers*** n (%)
P-gp	8 (13.6)	21 (35.6)	19 (32.2)	11 (18.6)	51 (86.4)
LRP	9 (15.3)	12 (20.3)	24 (40.7)	14 (23.7)	50 (84.7)
MRP	43 (72.9)	12 (20.3)	4 (6.8)	0 (0.0)	16 (27.1)

* r = 0.803, The expression of P-gp is correlated stong positively with LRP.

** *P *= 0.298, P-gp vs LRP.

*** *P *= 0.000, P-gp vs MRP; *P *= 0.000, LRP vs MRP; *P *= 1.000, P-gp vs LRP.

Pearson Chis-square test; Gamma test

### The relationship between the pathological types and the expression of P-gp, LRP and MRP

There were no statistically significant differences in the expressions of P-gp, LRP and LRP among different pathological types (*P *values are 0.561, 0.661 and 0.297, respectively). No significant difference between the expression of P-gp and LRP in poorly differentiated adenocarcinoma were observed (*P *= 0.716), but we showed a low positive correlation between them (r = 0.376) (Table 2).

**Table 2:** The expression of P-gp, MRP and LRP in patients with gastric cancer of different pathological types

		Positive rates of MDR proteins^b^		
		
Pathological types^a^	Numbers	P-gp* n (%)	LRP** n(%)	MRP*** n(%)
Poorly differentiated adenocarcinoma^#^	18	16 (88.9)	17 (94.4)	6 (33.3)
Moderately differentiated adenocarcinoma^##^	23	18 (78.3)	18 (78.3)	3 (13.0)
Well differentiated adenocarcinoma ^###^	8	7 (87.5)	7 (87.5)	4 (50.0)
Mucous adenocarcinoma	6	6 (100)	5 (83.3)	2 (33.3)
Others ^c^	4	4 (100)	3 (75.0)	1 (25.0)

^a ^Comparison between the expression of P-gp and LRP in the same pathological types:

^#^:*P *= 0.716; r = 0.376

^##^:*P *= 0.915; r = 0.913

^###^:*P *= 0.686; r = 0.414

^b^Comparison among different pathological types for the same protein:

*P = 0.561

**P = 0.297

***P = 0.661

^c^Others included well differentiated squamous carcinoma one case, unknown pathological types 3 cases.

Pearson Chis-square test and Gamma test

### The relationship between clinico-pathological stages and the expression of P-gp, MRP and LRP

P-gp was positively correlated with clinical stages (r = 0.742). There was a trend towards more advanced clinical stages with higher P-gp positive rate, although the differences among different clinical stages were not statistically significant (P = 0.304). The differences of LRP and MRP among different clinical stages were not statistically significant (P = 0.087 and 0.380, respectively) (Table 3).

**Table 3:** The relationship between clinico-pathological stages of gastric cancer and P-gp, MRP and LRP

		Positive rates of MDR proteins		
		
Stages	Numbers n(%)	P-gp* n(%)	MRP n(%)	LRP n(%)
TNM stages				
T2	13 (22.0)	12 (92.3)	6 (46.2)	10 (76.9)
T3	44 (74.6)	37 (84.1)	10 (22.7)	39 (88.6)
T4	2 (3.4)	2 (100)	0 (0.0)	1 (50.0)
N0	24 (40.7)	21 (87.5)	10 (41.7)	21 (87.5)
N1	18 (30.5)	14 (77.8)	2 (11.1)	15 (83.3)
N2	15 (25.4)	14 (93.3)	3 (20.0)	12 (80.0)
N3	2 (3.4)	2 (100)	1 (50.0)	2 (100.0)
M0	57 (96.6)	49 (86.0)	16 (28.1)	49 (86.0)
M1	2 (3.4)	2 (100.0)	0 (0.0)	1 (50.0)
Clinical stages				
IB	10 (16.9)	10 (100)	6 (60.0)	9 (90.0)
II	13 (22.0)	10 (76.9)	4 (30.8)	11 (84.6)
IIIA	18 (30.5)	14 (77.8)	2 (11.1)	16 (88.9)
IIIB	14 (23.7)	13 (92.9)	3 (21.4)	12 (85.7)
IV	4 (6.8)	4 (100)	1 (25.0)	2 (50.0)

* The positive rate of P-gp is correlated positively with clinical stages (r = 0.742).

## Discussion

Chemotherapy is an important treatment option in the multi-disciplinary treatment strategy against GC. It has been established that postoperative chemotherapy could help reduce the recurrence and improve the progression-free survival in resectable GC [8-10] and even in metastatic GC [11]. Most patients, however, will ultimately experience relapse and treatment failure usually within 2-3 years after surgery. A major cause for such recurrence is the chemoresistance in GC, which results from several molecular mechanisms. Among these, drug efflux transporters are the most intensively studied molecular families, including ATP-binding-cassette (ABC transporter) [12], which uses ATP to pump drugs out of the target cell and reduce the intracellular drug concentrations leading to drug resistance. Two members of the ABC transporter superfamily including P-gp and MRP play a major role in resistance [13]. Lung resistance protein (LRP) is a member of the vault proteins involved in MDR. LRP has been shown to shuttle anthracyclines out of the nucleus [14].

The expression of P-gp, MRP and LRP are positively correlated with the level of drug resistance. The assessment of MDR proteins over-expression is useful in determining the most appropriate chemotherapy regimen for GC. However, the positive rates of P-gp, MRP and LRP reported in the literature are variable. Alexander et al. [15] found by immunohistochemistry that the positive rates of MRP, LRP and P-gp were 55%, 10% and 0%, respectively. Fan et al. [16] found by reverse transcription polymerase chain reaction (RT-PCR) in 50 GC patients that the mRNA expressions of MRP, LRP, and MDR1 were 12.0%, 10.0% and 10.0%, respectively. More recent studies [17-19] using immunohistochemistry found that the positive rates of MRP and LRP ranged from 39.4% to 88.9%. The positive rates of these three MDR markers in our 59 patients are higher compared with those results, probably due to improved detection technology.

Our study found no significant differences among the expressions of P-gp, MRP and LRP in GC of different pathological types, in agreement with findings by Shi et al [20], who found that the positive rates of P-gp and LRP were 49.2% and 58%, respectively, and such expression was closely related to clinicopathological staging but not related to tumor differentiation. In our study, MRP and LRP expression was not related to tumor invasion depth or lymphatic metastasis. Based on these findings, we propose that innate resistance may exist in those 59 GC patients even without prior chemotherapy.

P-gp confers resistance to cytotoxicity by chemotherapy drugs, cytokine TNF-alpha, and ultraviolet light [21]. Faggad et al. [22] found that MRP1 expression was as an independent negative prognostic factor for overall survival in ovarian cancer. As the patients in our group had mixed postoperative treatment, it is impossible to correlate these findings with clinical outcomes. This is the limitation of the current study, and future work should be done to elaborate on this issue.

The expression of P-gp, MRP and LRP confers different drug resistance profiles [23], including P-gp conferring resistance to doxorubicin, vincristine, vinblastine, actinomycin-D and paclitaxel, MRP conferring resistance to etoposide and epirubicin, and LRP conferring resistance to carboplatin and Melphalan. Our study found these molecules are interrelated, and P-gp is correlated with LRP (r = 0.803), especially for moderately differentiated adenocarcinoma (r = 0.915). The finding suggests that both two resistance mechanisms exist in most patients.

As the resistance mechanisms of P-gp, MRP and LRP are clarified, suggestions are proposed if we can block all the ABC transporters at once [24]? Recent studies revealed some new methods to overcome MDR, such as specific PI3K inhibitors to reduce P-gp [25,26]. Du [27] showed that RP L6 could regulate MDR in GC cells by suppressing drug-induced apoptosis. Robey [28] reported an initial phase I studies of CBT-1, an orally-administered, bisbenzylisoquinoline plant alkyloid as P-gp inhibitor. CBT-1 at 1 μM completely reversed P-gp-mediated resistance to vinblastine, paclitaxel and depsipeptide.

Although the value of systemic chemotherapy for GC is controversial, several studies have demonstrated that GC could benefited for chemotherapy [29], although MDR remains a major challenge to effective chemotherapy [30]. Combined determination of P-gp, MRP and LRP may help tailor the chemotherapy regimes and predict the outcomes of treatment.

## Conclusion

There are high percentages of innate expressions of P-gp, LRP and MRP in GC without prior chemotherapy, which may contribute to the poor response to chemotherapy of GC.

## List of abbreviations

GC: gastric cancer; MDR: multidrug resistance; P-gp: P-glycoprotein; MRP: Multidrug resistance-associated protein; LRP: Lung resistance protein.

## Competing interests

The authors declare that they have no competing interests.

## Authors' contributions

Hu WQ selects the research topic, participates in the study and provides partial grant support. Peng CW conducts the pathological examination, statistical analysis and writes manuscript. Li Y conceives the study project, organizes the whole study process, provides financial support, and finalizes the manuscript. All authors have read and approved the final manuscript.
